# Comparative Evolution of S7 Intron 1 and Ribosomal Internal Transcribed Spacer in *Coilia nasus* (Clupeiformes: Engraulidae)

**DOI:** 10.3390/ijms13033085

**Published:** 2012-03-07

**Authors:** Dong Liu, Hong-Yi Guo, Wen-Qiao Tang, Jin-Quan Yang

**Affiliations:** College of Fisheries and Life Science, Shanghai Ocean University, Shanghai, 201306, China; E-Mails: dliu@shou.edu.cn (D.L.); hy-guo@shou.edu.cn (H.-Y.G.); jqyang@shou.edu.cn (J.-Q.Y.)

**Keywords:** ribosomal protein S7 gene, internal transcribed spacer, *Coilia nasus*, comparative evolution, phylogeny, concerted evolution

## Abstract

*Coilia nasus* is widely distributed in the Yangtze River, the coastal waters of China, Korea and the Ariake Sound of Japan. Several ecotypes exist and this provides a useful model for the study of comparative diversity between molecular markers. Here we analyze and compare the nucleotide sequences between single-copy ribosomal protein S7 gene intron 1 (*rpS7*) and multiple-copy ribosomal internal transcribed spacer 1 (ITS1) in this species to compare the phylogenetic signal of the two nuclear genes. Nucleotide substitutions among the two gene sequences and partial sequence of mitochondrial cytochrome c oxidase subunit I (COI) gene were also analyzed. A total of 115 clones for *rpS7* and 122 clones for ITS1 were obtained from 37 specimens. The nucleotide sequence length is 741 to 743 bp for *rpS7* and 334 to 348 bp for ITS1. Intra- and inter-specimen variation in *rpS7* results from nucleotide substitution, while such variation in ITS1 is mainly due to different numbers of short base repeats. The content of G + C is lower in *rpS7* (43.5%) than in ITS1 (68.2%). Our results indicate that the proportion of the sequence variable sites is higher in *rpS7* (61) than in ITS1 (23); the informative parsimony of *rpS7* is evidently higher than that of ITS1 (26 *vs*. 2); the overall ratio between transitions and transversions in ITS1 is slightly lower than in *rpS7*, but remarkably lower than in COI. These results suggest that *rpS7* is more suitable than ITS1 as a marker for genetic divergence of this group. Furthermore, gene flow is observed between the different geographic populations of *C. nasus* from the phylogeny of this species based on *rpS7*, showing that *rpS7* has more evolutionary characteristics for understanding the processes of genomic evolution at the intraspecific level.

## 1. Introduction

Well-selected molecular markers are crucial for the identification and phylogenetic study of closely related species, and within species with different geographic distributions. Such markers can aid in understanding the processes of biological diversification. One useful marker is the *rpS7* (ribosomal protein S7) gene, which consists of six introns and seven exons [1]. The first two introns of *rpS7* show a high rate of evolution, less base compositional bias, and a relatively low transition/transversion ratio. The evolutionary characteristics of this nuclear marker have been used for systematic studies at the subfamilial level in African electric fishes [2]. By providing more information in sequence variation than the second one, the first intron is widely used as a marker for molecular phylogeny in cyprinid fishes [3], cichlid fishes [4], and darter fish genus *Etheostoma* [5]. The *rpS7* gene is a single-copy gene and the conserved stretches of DNA in its adjacent exons have been used to design primer pairs for amplification of the intronic regions in fish which are more likely to be variable [6]. The *rpS7* gene coupling with other nuclear and mitochondrial genes has been used as markers to investigate molecular phylogeny in various organisms [2,4,7]. The mitochondrial genome is maternally inherited and thus does not permit the detection of historical hybridization events resulting from the sexually reproducing organisms. However, the *rpS7* alleles have a biparental mode of inheritance, and the allelic recombination enables the detection of such events [8,9]. However, Keck *et al*. [7] reported that the *rpS7* dataset lacked the resolution required to determine interspecific relationships of *Nothonotus* darters, but was more useful for characterizing intraspecific relationships. Guo *et al.* [10] found that *rpS7* has more informative parsimony than *cty b* in sinipercid fishes, although little is known about the intraspecific diversity of the *rpS7* gene.

Another widely used marker is the first ribosomal internal transcribed spacer (ITS1), which is one region of the ribosomal RNA (rRNA) gene. Multiple copies (100–500) of the rRNA gene cluster in tandem, and each transcription unit is composed of 18S rRNA coding region, ITS1 region, 5.8S rRNA coding region, ITS2 region and 28S rRNA coding region [11]. The ITS1 region is one of the most variable parts of the genome, able to verify and align sequences according to the conserved parts of rRNAs. Therefore it is suitable for phylogenetic analyses of closely related species [12]. The polymorphism studies of ITS1 in various organisms indicate concerted evolution among the products of this gene duplication, a process of homogenization involving reciprocal and nonreciprocal recombination for ribosomal DNA within and between chromosomes [13,14]. It results in interspecific divergence and intraspecific similarity among each copy of ITS1 [13]. The hypothesis of concerted evolution has been demonstrated in *Cicindela dorsalis* [14], crayfish species [15] and brown trout *Salmo trutta* [16]. Recently, the putative secondary structure of ITS1 was successfully used for species identification, as well as phylogenetic reconstruction of taxonomic groups in plants, insects and animals [17–19].

**Figure 1:**
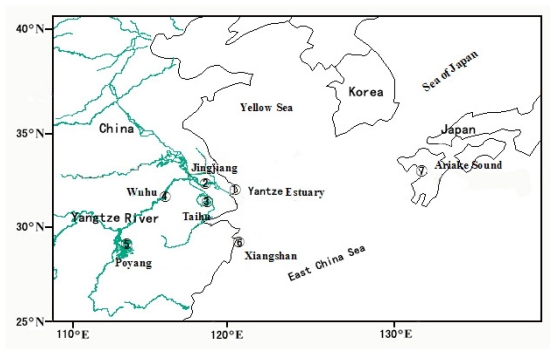
Map of the *Coilia nasus* collection sites. The numbers denote 1 as Yangtze estuary (YE), 2 as Jingjiang (JJ), 3 as Lake Taihu (TH), 4 as Wuhu (WH), 5 as Lake Poyang (PY), 6 as Xiangshan (XS), and 7 as Ariake Sound (AS).

The grenadier anchovy *Coilia nasus* is a small-to-moderate-sized fish, widely distributed in the Yangtze River, localized in the coastal waters of China, and Korea as well as the Ariake Sound of southwestern Japan [20]. Earlier morphological and ecological studies of the grenadier anchovy have indicated that it represents a species complex, composed of *Coilia nasus*, a species that displays homing behavior, a closely related species *Coilia brachygnathus*, and one subspecies *Coilia nasus taihuensis* [21]. The homing species is an anadromous fish. The sexually mature fish migrate for spawning from their oceanic habitat to the Yangtze River during the reproductive season (March to August). After reproduction, these fish and their progeny then migrate back to the ocean in September to November of the same year. The latter two species inhabit only freshwater. *C. brachygnathus* spawns and lives in the middle and lower reaches of the Yangtze River. *C. nasus taihuensis* is only found in Lake Taihu, a landlocked freshwater lake in China (Figure 1). *C. brachygnathus* can be distinguished morphologically from *C. nasus* by its relatively reduced upper jaw length. *C. nasus taihuensis*, on the other hand, is distinguished from *C. nasus* by its reduced number of vertebrae [20]. Recent studies suggest that *C. nasus taihuensis* and *C. brachygnathus* are indistinguishable from *C. nasus* based on mitochondrial sequence and otolith characters [22,23]. Now the subspecies and the related species are regarded as the ecological forms of *C. nasus* throughout its distribution [24]. These ecotypes of *C. nasus* have high genetic diversity on mitochondrial markers, but they show limited population genetic structure [25].

In this study, we have analyzed *rpS7* and ITS1 in 37 specimens of the grenadier anchovy *C. nasus* collected from seven geographical populations to investigate the sequence diversity and to compare evolutionary characteristics of the two ribosomal genes at the intraspecific level. In doing so, we selected a partial sequence of mitochondrial cytochrome c oxidase subunit I (COI) gene, the so-called “DNA barcode”, as a standard for assessing the overall ratio between transitions and transversions of the nucleotide sequence. We selected COI for our analysis, also due to its widespread and successful utilization in discriminating sister species [26], hence we applied this approach to identify representative samples of the *C. nasus* complex. To our knowledge, the present study is the first report to compare molecular diversity between the single-copy gene *rpS7* and the multiple-copy gene ITS1 within species. Our results indicate that considerable intragenomic variability exists in *rpS7* due to mutation, while ITS1 contains different numbers of repeat motifs that occupy the internal loops of the putative secondary structure. Trees constructed with *rpS7* show a stronger phylogenetic signal than ITS1 based on the *C. nasus* dataset, making *rpS7* a preferred choice for population studies in this species.

## 2. Results and Discussion

### 2.1. Results

#### 2.1.1. Composition and Variation of *rpS7* Intron 1

A total of 115 *rpS7* clones were sequenced for 37 *C. nasus* individuals from seven sampling sites (Figure 1), yielding intron fragments that were 741–743 bp long. In total 29 different haplotypes were identified. Sequences were deposited in GenBank under accession numbers JN394513-394541. Haplotype alignments and their frequencies were shown in Table 1. Their codes include the abbreviation of the locality names in Figure 1, then an Arabic numeral (which denoted the specimen number for each sampling site), and a lowercase numeral (in the cases where there were multiple haplotypes for each individual). One common haplotype (H29) was shared by eight clones (TH5a, YE3a, XS1a, XS2a, XS3, JJ6a, WH3 and AS), producing from these specimens sampling from six localities (TH, YE, XS, JJ, WH and AS). Three haplotypes (H12, H13 and H28) were encountered twice (PY3b and YE4; PY4 and YE3b; JJ6b and WH4). Ten out of the thirty-seven *C. nasus* individuals examined showed two different sequence types with variable positions (3–16), in which three individuals (PY3, TH6 and YE3) were found a 1-bp gaps and missing (Indel), and three individuals (TH1, XS1 and XS2) were observed a 2-bp Indel, and one individual (JJ5) was detected a 1-to 2-bp Indel (Table 1). Four of the individuals in our study displayed two alleles, which may have been the result of intraspecific hybridization events (Table 2). For example, position 52 was a putative parental allele, because (1) the T nucleotide was present in 21 clones from five individuals of TH population; (2) the A nucleotide was present in 25 clones from six individuals of PY population; (3) the heterozygosity of the two nucleotides were found in the individuals from JJ and SX populations. However, one concern of molecular variation at the intragenomic level is the reliability of the amplification reaction, in which DNA stretches leads to copying errors by *Taq* polymerase in a way analogous to the biological replication process [27]. In our study, these position nucleotides could not be errors produced by *Taq* enzyme, because nucleotide positions did not vary in fifteen clones from five individuals of the AS population; this population was an isolated one in comparison of the other six, could be regarded as ancestral parent (Table 2). The nucleotide compositions and variable information of 29 haplotypes were shown in Table 3.

Testing nucleotide recombination detected three recombination positions (*R*_m_ = 3), and analysis of nucleotide heterogeneity indicated that three clones (JJ1a, PY2 and TH1a) were possibly involved recombination events (*P* < 0.05). These putative recombination events were omitted from the following analysis. The average ML distance was 0.014 for the overall population. Specimens from four populations (JJ, YE, XS and WH) that traditionally represent *C. nasus* were treated as a compound population (CP). The ML distance between PY and TH/AS was 0.016/0.014, while the highest ML distance of 0.017 between CP and TH was more than four times that of the lowest distance between TH and AS (0.004); this suggests that TH and AS populations were entirely isolated populations. Analysis of nucleotide diversity among populations showed that the value between JJ and TH was the highest, and that between PY and TH was the lowest (Table 3). Statistical testing of the *rpS7* neutrality gave values of −1.328 (Tajima’s D) and −2.377 (Fu), although there was no significant difference (*P* > 0.1 for each). AMOVA, neutrality tests, and the rate of the nucleotide substitution were described in Table 3. Nucleotide substitution favored transitions over transversions, and the relative rate of nucleotide substitution was 2.8 (Table 3). A plot of transitions and transversions *versus* genetic distance showed that the index of substitution saturation (*ISS*) was 0.0173, *ISS* < *ISS.C* (critical value 0.7474), and the interpretation result was significantly different. Therefore, it can be concluded that the *rpS7* gene did not reach saturation, and this gene dataset can be used to restructure the phylogenetic relationship of the *C. nasus* complex.

#### 2.1.2. Compositional Information of ITS1

For each of the 37 *C. nasus* individuals 3–6 clones (total 122 clones) were isolated and sequenced, which produced 29 haplotypes. Sequences were deposited in GenBank under accession numbers JN394484-3944512. One common haplotype was encountered in all seven populations (frequency = 29.5%). Excluding rRNA coding regions, the aligned ITS1 sequence of *C. nasus* ranged from 334 to 348 bp. Overall, the number of variable positions was low (Table 3). Two parsimony positions (75: T/C and 76: T/G) were found between individuals PY3 and WH1 (These codes referred to that of *rpS7* mentioned above), and also within individuals. For some individuals, 2–4 different clones were observed, and the sequences of the clones differed by at least one position. Two types of tandem repeat, a 2-bp (CT) repeat and a 5-bp (CCAAA) repeat (Table 4), were detected to be variable for duplicated numbers within inter- and intra-individual, resulting in the variability of ITS1 in length. Tests of base homogeneity were congruent. Base pair composition, positions of nucleotide variability, and rate of transitions and transversions were shown in Table 3. The relative rate of nucleotide substitutions was 2.1 (Figure 3). AMOVA showed that nucleotide diversity within populations (89.7%) was significantly higher than that between populations (10.3%) (Table 3).

The ITS1 RNA secondary structure featured eight hairpin loops, one lateral loop and six internal loops (abbreviated IL) (data not shown, and folding temperature was fixed at 37 °C, initial ΔG = −166.30 kcal/mol). The shortest stem in the structure was 4 bp, while the longest stem was 22 bp. Approximately 80% of ITS1 nucleotides were involved in the formation of hairpin loops. By comparing the secondary structure and the primary aligned sequences, it revealed variations for mostly indel sites on the IL-2 and IL-4 regions. A 5-bp repeat was duplicated in the IL-2 region, and different numbers of a 2-bp repeat were found in the IL-4 region (Table 4); these repeats resulted in the variation of ITS1 length. 15 out of 23 variable positions were found in loop regions (including hairpin, lateral and internal loops), and eight were found in the stem regions where four were compensating base changes (CBCs). The variation in the primary sequence of the stem region did not disturb the base pairs, suggesting CBCs in the stem regions must be frequent to maintain functionality in the secondary structures.

#### 2.1.3. Phylogenetic Analysis of *rpS7* Gene

**Figure 2:**
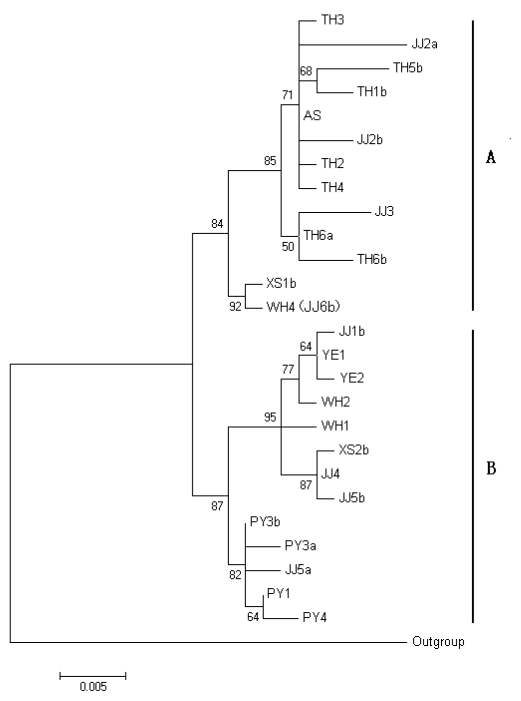
The Maximum-likelihood tree resulted from *rpS7* intron 1 sequence. Numbers beside the nodes stand for bootstrap values from 1000 replicates. The abbreviations represent site, including TH (Lake Taihu), JJ (Jingjiang), AS (Ariake Sound), XS (Xiangshan), WH (Wuhu), YE (Yangtze estuary), and PY (Lake Poyang). Arabic numerals denote specimen number, and lowercases indicate haplotyes per individual. *Coilia grayii* was used as an outgroup.

The genus *Coilia* composed of three species in China, *C. nasus*, *C. grayii* and *C. mystus* [20]. By utilizing the complete control region sequence of mitochondrial DNA (mtDNA) to investigate phylogenetic relationships of this group, *C. grayii* was found to be the sister group to *C. nasus*, and *C. mystus* occurred in the root of the phylogenetic tree [25]. Hence, *C. grayii* was used as a potential outgroup in this study. A phylogenetic tree of *C. nasus* was obtained from the *rpS7* dataset by MEGA 5.0 with Maximum-likelihood (ML) analysis (Figure 2). The tree was supported by the bootstrap test (≥50%). In the tree presented in Figure 2, the haplotypes of *rpS7* formed two major clades. Clade A was divided into two subclades. The larger subclade included the migratory (JJ) and landlocked freshwater ecotypes (TH); the small subclade included the migratory (WH) and marine ecotypes (XS). Clade B consists of two subclades. One subclade composed of freshwater (PY) and migratory ecotypes (JJ); the other subclade showed a complex of marine (XS) and migratory ecotypes (YE, JJ and WH). It was worth noting that the subclade in clade A composed of XS1b and WH4; according to the two facts, (1) one common haplotype (H28) was shared by WH4 and JJ6b (Table 1); and (2) XS1 and JJ6 have a varying nucleotide at four positions (Table 2); hence the subclade showing putatively gene flow could be potentially detected by the *rpS7* phylogenetic tree.

#### 2.1.4. Mitochondrial COI Molecular Variety

**Figure 3:**
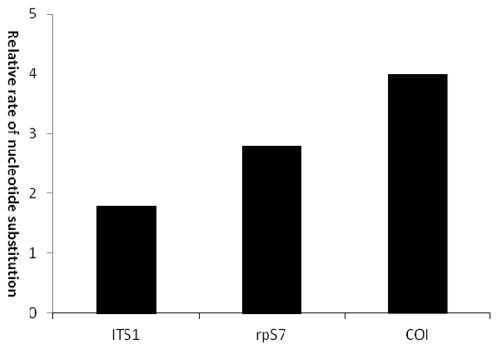
The relative rates of nucleotide substitution for nuclear and mitochondrial genes in *Coilia nasus*.

The mitochondrial barcode derived from COI is regarded as a standard reference of diversity in DNA sequence. This marker has been used extensively for species identification. COI fragments of 652 bp in length were sequenced for 37 *C. nasus* individuals—a total of 9 haplotypes were detected. Their sequences have been deposited into GenBank (JN394472-394480). The base composition, *etc*. were described in Table 3. The average K2P distance-based COI was 0.2–0.4% (intrapopulation) and 0.1–0.5% (interpopulation). If the specimens of *C. brachygnathus* and *C. nasus taihuensis* were identified according to the previous morphological standard [21], and the representative specimens were treated either as separate species/subspecies or as single species, then the average K2P distance was 0.2–0.4% (intraspecies) and 0.3–0.6% (interspecies). These results revealed that the value of molecular divergence in *C. nasus* was far lower than the 2% sequence divergence that represents congeneric species of fish [28]. When datasets of COI, ITS1 and *rpS7* from *C. nasus* were compared, the number of parsimony informative sites of the first two (6 and 2, respectively) was significantly lower than the latter (26); the nucleotide diversity of COI (0.003) was roughly equal to that of ITS1 (0.006), but both them were evidently lower than that of *rpS7* (0.013) (Table 3). When the relative rate of nucleotide substitutions (transitions/ transversions) among the three genes was compared, ITS1 was slightly lower than *rpS7*, but remarkably lower than COI (Figure 3). These results indicate that *rpS7* have more sequence diversity than ITS1 or COI, and the nuclear *rpS7* marker could be phylogenetic utility of this group at intraspecific level.

### 2.2. Discussion

The *C. nasus* complex exhibits morphological and ecological divergence across its natural distribution, and represents an ideal model in which to investigate the intraspecies diversity of nuclear sequences for *rpS7* and ITS1. According to our study on 812 specimens covering the entire species complex of *C. nasus* collected from the middle and lower regions of the Yangtze River, local populations could be distinguished from one another on the basis of morphological characters such as maxillary length, vertebrae number and anal fin ray number, although identification characters have overlapped in some specimens [29]. The first 648 bp of COI has been widely used as a DNA barcode, an approach that has proven particularly useful for discriminating ~5000 sibling species [26]. The COI sequence of *C. nasus* indicates the molecular divergence at the level of species does not reach the threshold of 2% that represents sibling species [28], thus the representative specimens covering the *C. nasus* complex from several populations provide a comparative clue for the divergence of *rpS7* and ITS1 at the intraspecific level.

The length of *rpS7* in *C. nasus* is around 700 bp, which is similar to other fish, e.g., Sinipercid fish [10] and Notothenioid fish [30]. The length of the aligned ITS1 gene in *C. nasus* is about 350 bp, shorter than that of *Salvelinus* fish (596 bp) [31] and *Salmo trutta* (582 bp) [16]. The numbers of parsimony positions in ITS1 was lower (2) than in *rpS7* (26), indicating that the divergence rate of the sequences differed between the two nuclear genes; furthermore, the results of the nucleotide diversity analysis and the value for transitions proportional to transversions also supported this hypothesis (Table 3). In other studies, the first intron of *rpS7* gene is 719 bp long, of which 168 positions are parsimony informative in 40 mormyroid species [2]; ITS1 from six species of the salmonid fish genus *Salvelinus* is 596 bp long, in which 45 are informative sites based on six sequence alignments [31]. For the intraspecies variation of ITS1 among 86 brown trout individuals by DNA directly sequencing, only 16 informative parsimony sites are found [16].

The evolutionary characteristics of the single-copy nuclear *rpS7* gene are a model of biparental inheritance [2], and the putative hybridization between parents could potentially be detected with parsimony information of *rpS7* [9]. In our surveyed populations, *rpS7* has aided in the potential detection of hybridization events. The putatively intraspecific hybrid individuals, *i.e.*, JJ1, JJ6, XS1 and XS2 are polymorphic at four positions of *rpS7*, while the contributing parents (TH and PY) show fixedly single nucleotide differences at these four sites (Table 2). The conclusion from morphologically characteristic data is that the putative hybrid specimens possess an intermediate upper jaw length between TH and PY specimens [29]. In addition, TH and AS samples maybe represent two potentially ancestral parents because they are geographically isolated populations but share the same haplotypes. The two geographical populations were produced by climatic oscillations during the Pleistocene ice ages [25]. Lake Taihu is adjacent to the Yangtze River, and TH population occasionally exchange with other geographical populations during flooding. In general, PY samples are characterized by reduced upper jaw length [20]. The Poyang Lake merges with the Yangtze River. It facilitates the intraspecific hybrid events to occur between PY population and other geographical populations. Therefore, intraspecific hybridization events might be occurring in *C. nasus* and such events could potentially be detected with *rpS7*. The high diversity of *rpS7* sequences within and between individuals is consistent with various phenotypes in the *C. nasus* complex from several populations. The greatest variation of *rpS7* in specimens from the middle and lower regions of the Yangtze River was due to recombination events; it is known that recombination can give more sequence divergence than mutation [32]. Our nucleotide sequence-based study provides evidence that the *rpS7* could be used as a useful molecular marker for our study group.

In contrast, ITS1 indicates concerted evolution among the products of this gene duplication, resulting in interspecific divergence and intraspecific similarity among each copy [13]. The effects of concerted evolution usually impede the possibility to determine whether individuals are heterozygous or homozygous [33]. ITS1 shows concerted evolution and significantly low level of divergence among alleles in our study, and the varying number of repeat motifs results in different lengths of the ITS1 variants (334–348 bp). The considerable intragenomic variation in the number of repeat elements of ITS1 is enough to obscure phylogenetic relationships of crayfishes at the population level [15]. Nevertheless, the sequence divergence in ITS1 of tiger beetle *Cicindela dorsalis* shows that the variation within individual was almost as high as the variation within the entire lineage based on a total of 50 clones obtained from 12 specimens [14]. The repetitive ITS1 could provide genetic markers appropriate for population structure of scleractinian corals and sponges at inter- and intra-specific levels [33]. When ITS1 was used as a molecular marker for phylogeographic analysis of brown trout *Salmo trutta*, the results were congruent between both ITS1 and mtDNA [16]. Based on previously published data, it can be difficult to predict whether ITS1 will be suitable for phylogenetic analysis at the intraspecific level in a given taxon.

The intra- and inter-individual variation in *rpS7* could be seen to have occurred via nucleotide transitions and transversions, while the variation in ITS1 was mostly due to varying numbers of indels (Table 3). The relative rate of nucleotide substitutions in the *rpS7* sequence was slightly higher than that of ITS1, but remarkably lower than that of COI (Figure 3). Similarly, the overall ratio between transitions and transversions in *rpS7* is lower than in *cyt b* in Sinipercid fishes [10]. Comparison of the populations found that the molecular variance in ITS1 was ~three times lower (10.3%) than that of *rpS7* (29.9%) (Table 3). The results demonstrate the concerted evolution of ITS1, similar to the lower evolutionary rates for ITS1 reported in brown trout *Salmo trutta* [16] and truffle *Terfezia terfezioides* [34]. Although different numbers of repeat elements producing sequence differences were encountered in ITS1 within and between individuals (Table 4), these repeat elements were observed in the internal loops of the predicted secondary structures, a major portion of the transcript in which the repeat element facilitates insertions and deletions by a slipped-strand mispairing mechanism [35]. Various patterns of repeat motifs have been published [13–15], yet to our knowledge, this is the first report of a “CT” repeat and different numbers of the repeat motif found in the internal loops that did not disturb functionality in the secondary structures.

The phylogenetic tree, restructured through maximum-likelihood method, was partially congruent with previous mtDNA analysis [25]. The samples from the four sites (PY, JJ, TH, and XS) grouped into two main lineages, but such lineages did not correlate with geographical populations based on the complete control region sequence of mtDNA [25]. In contrast, *rpS7* was better able to resolve population relationships than mtDNA, e.g., all samples from TH, so-called *C. nasus taihuensis*, could be grouped into one subclade, and all samples from PY, so-called *C. brachygnathus*, formed another subclade, although the two subclades mixed the individuals sampled from other geographical populations. Furthermore, the proportion of the bootstrap values of the two major lineages was evidently higher in *rpS7* (84%) than in mtDNA (50%). Interestingly, one subclade composed of XS1b and WH4 (Figure 2); the two haplotypes represent one of the varying genotypes, respectively (WH4 and JJ6b shared haplotype H28) (Table 2). Therefore, this subclade has potentially aided in the detection of a putative gene flow. This result indicates that gene flow maybe occurs among different ecotype of *C. nasus*, providing a clue for understanding the processes of its genomic evolution.

## 3. Experimental Section

### 3.1. Sample Collection

Specimens were collected from five sites from the middle and lower reaches of the Yangtze River, represented by the following codes: Yangtze estuary (YE), Jingjiang (JJ) in Jiangsu province, Wuhu (WH) in Anhui province, Lake Poyang (PY), and Lake Taihu (TH). Two further sites were Xiangshan harbor (XS) in the coastal region of the East Sea in China, and the Ariake Sound (AS) in Japan (Figure 1). *C. nasus* were sampled across the seven field sites, which enabled the inclusion of all ecotypes of this species. Specifically, YE, JJ, WH and PY samples represent the freshwater populations of either *C. brachygnathus* (collected in May to June) or *C. nasus* (collected in December to January); TH samples represent the landlocked ecotype of *C. nasus taihuensis*; XS samples represent the marine ecotype of *C. nasus*. The isolated AS samples were used as a reference. A total of 812 specimens collected from the seven sites were used to identify species according to the morphological characters [29], and 37 (5–6 from each site) were randomly chosen as representative samples, analyzed by DNA sequencing for *rpS7*, ITS1, and COI genes. Muscle tissue that was used for molecular analysis was preserved in 95% ethanol, and the specimens were preserved in 70% ethanol and deposited in the Fish Collection of Shanghai Ocean University in China.

### 3.2. DNA Extraction, PCR Amplification, Cloning, and Sequencing

Total genomic DNA was extracted from small amounts of ethanol-preserved muscle by proteinase K digestion in lysis buffer at 55 °C for 2–3 h, from the DNA extractor kit according to the manufacturers’ instructions (Sangon, China). PCR primer sequences are listed in Table 5. Primer sequences for *rpS7* were obtained as described previously [6]. Primers for ITS1 and COI were designed using Primer Premier, version 5.0 [36], based on the sequences published in the National Centre for Biotechnology Information (NCBI) database. PCR reactions were performed in 50 *μ*L mixture contained 100 ng of genomic DNA, 0.2 *μ*M of each primer, 50 *μ*M of each dNTPs, 5 *μ*L of 10× plus buffer, and 2U *Taq* plus polymerase (Tiangen, Beijing, China). PCR amplification was carried out in an Eppendorf Mastercycler (Eppendorf, Munich, Germany) as follows: 94 °C for 3 min, and 30 cycles of 94 °C for 30 s, 58 °C for 30 s (52 °C for ITS1, 56 °C for COI), and 72 °C for 1 min. A final extension was performed at 72 °C for 10 min. Amplification products were separated, purified and transformed into *E. coli* DH5*α* cells as described [13] except that the purified products of COI were directly sequenced. Positive clones were identified by blue/white screening. For sequence variation analysis at intraspecies level, 3–6 clones/specimen were randomly chosen and sequenced as described [13]. To be sure the sequence accuracy, all the clones were bidirectionally sequenced with vector-specific primers M13 and SP6 by an automated DNA sequencer (ABI PRISM 3730).

### 3.3. Molecular Data Analysis

Sequence identity was confirmed based on the published sequences in the NCBI database. DNA sequences were aligned through a multiple sequence alignment with the ClustalX2 program, version 2.0 [37]. The ITS1 secondary structures were generated as described [38]. After structural elements were identified in the transcripts, the alignment was refined and screened for compensating base changes. The folding pattern of secondary structure elements was predicted on the web Mfold server as described [39]. Nucleotide composition, variable sites, position recombination, number of haplotypes and haplotype diversity were calculated using DnaSP software, version 5.10 [40]. MEGA software, version 5.0 [41] was used to calculate pairwise distances with the maximum composite likelihood method, the homogeneity of the base composition with the Id-test, the average Kimura 2 parameter (K2P) distance, and the relative rates of nucleotide substitution. Population genetic structure was evaluated by the analysis of molecular variance (AMOVA) method [42] using Arlequin, version 3.5 [43]. The possibility of substitution saturation was investigated by DAMBE software, version 5.2 [44] using the presence of nucleotide substitutions *versus* maximum likelihood distance. Neutrality of population genetic variation was estimated using Tajima’s D and Fu’s Fs statistical tests, both methods were implemented by the DnaSP software [40]. The arrays of phylogenetic analyses were performed by a maximum- likelihood approach in MEGA 5.0 [41]. The bootstrap values for the maximum likelihood tree were estimated using searches with 1000 replicates.

## 4. Conclusions

Results from this study are likely to represent the first data on comparison of different nuclear DNA markers *rpS7* and ITS1 for estimating intraspecific genetic diversity in genome of *C. nasus*. The *rpS7* exhibits more phylogenetic signal than ITS1, and can be considered as a useful molecular marker required in detection of potentially intraspecific hybridization events, as well as for phylogenetic analysis of this taxon. In contrast, ITS1 shows a pattern of concerted evolution and sequence homogenization among the products of this gene duplication at intraspecific level.

## Figures and Tables

**Table 1:** Alignment, frequencies and haplotypes of *rpS7* in *Coilia nasus*.

	Sequence	Frequency	Haplotype
	
Code	0000000001111111112222222233333344444444444555555555555566666666677777		
	5555577880015568990113459934566912233668899000011233456900113344812234		
	2567856171930927561485444627616220556057938346907548233347461801121943		
JJ1a	tgtggattttacttcgacttgttaatttacccaagatatacattaggattgctctatactaaacacttga	2	H1
JJ1b	a a . . . . c . . . . a . . t . . . . . . . . . . c . . . g . . . . . c . g . . . . . g . a . . . . . . a . c . . . . . . . . . g . . . c .	3	H2
JJ2a	. . . . . c . . . . . . . . . . . . . . . c . . g . . . . . . . . . . . . . . . . . . . . . . . c . . . . . . . . . . . . . . . g . . c c .	1	H3
JJ2b	. . . . . . . . . . . . . . . . . . . . . . . . . . . . . . . . . . . . . . . . . . . . g . . . . . . . . . . . . c . . . g . . g t . . c .	2	H4
JJ3	. . . . . . . . . . . . . c . . . . . . . . a . . . . . . . t . . . . c c . . . . . . . . . . . . . . . . . . . . c t . . . . . g . . . c .	3	H5
JJ4	a a . . . . c . . . . a . . t . . . . . . . . . . c . . . . . . . . . c . g . . . . . g . a - - . . . . . t . . . . . . . . . t g . . . c .	2	H6
JJ5a	a . . . t . . . . . . a . . . . . . . . . . . . . c . . . . . - . . . c . g . . . g . g . a . . . . . . . . . . . . . . . . . . g . . . c g	2	H7
JJ5b	a a . . . . c . . . . a . . t . . . . . . . . . . c . . . . . . . . . c . g . . . . . g . a - - . c . . . t . . . . . . . . . t g . . . c .	2	H8
PY1	a . . . t . . . . . . a . . . . . . . . . . . . . c . . . . . - . . . c . g . . . . . g . a . . . . . . . . . . a . . . . . . . g . . . c .	3	H9
PY2	a a . . . . c . . a . a a . t . . . c c . . . . . c . c . g . . . . . c . g . . . . . g . a . . . . . . . . c g . . . . . . . . g . . . c .	2	H10
PY3a	a . . . t . . c . . . a . . . . . . . . . . . . . c . . . . . - . . . c . g . . . . c g . a . . . . . . . . . . . . . . . . - . g . . . c .	2	H11
PY3b	a . . . t . . . . . . a . . . . . . . . . . . . . c . . . . . - . . . c . g . . . . . g . a . . . . . . . . . . . . . . . . . . g . . . c .	5	H12
PY4	a . . . t . . . . . . a . . . . . . . . . . c . . c . . . . . - . . . c . g . . . . . g . a . . . . . . . . . . a . . c . . . . g . . . c .	6	H13
TH1a	. . - - . . . . . . . . . . . . . . . . a . . . . . . . . . . . . . . . . . . . . . . . . . . . . . . . . . . . . c . . . . . . g . . . c .	2	H14
TH1b	. . . . . . . . . . g . . . . . . . . . . . . . . . . . . . . . . . . . . . . . . . . . . . . . . . . t . . . . . c . . g . . . g . . . c .	1	H15
TH2	. . . . . . . . . . . . . . . . . . . . . . . . . . . . . . . . . . . . . . . . . . . . . . . . . . . . . . . . . c . . . . . . g . c . c .	3	H16
TH3	. . . . . . . . . . . . . . . . . . . . . . . . . . . . g . . . . . . . . . . . . . . . . . . g . . . . . . . . . c . . . . . . g . . . c .	3	H17
TH4	. . - - . . . . . . . . . . . . . . . . . . . . . . c . . . . . . . . . . . . . . . . . . . . . . . . . . . . . . c . . . . . . g . . . c .	5	H18
TH5b	. . . . . . . . c . g . . . . . . . . . . . . t . . . . . . . . . . . . . . . g t . . . . . . . . . . . . . . . . c . . . . . . g . . . c .	2	H19
TH6a	. . . . . . . . . . . . . . . . . . . . . . . . . . . . . . . . . . . c . . . . . . . . . . . . . . . . . . . . . c t . . . . . g . . . c .	3	H20
TH6b	. . . . . . . . . . . . . . . - . . . . . . . . . c . . . . . . . . . c . . c . . . . . . . . . . . a . . . . . . c t . . . . . g . . . c .	2	H21
YE1	a a . . . . c . . . . a . . t . . . . . . . . . . c . . . g . . . . . c . g . . . . . g . a . . . . . . . . c . . . . . . . . . g . . . c .	3	H22
YE2	a a . . . . c . . . . a a . t . . . . . . . . . . c . . . g . . . . . c . g . . . . . g . a . . . . . . . . c . . . . . . . . . g . . . c .	3	H23
XS1b	a . . . . . . . . . . a . . . . - - . . . . . . . c . . . . . . g . . c . . . . . . . . . . . . . . . . . . . . . c . . . . . . . . . . c .	2	H24
XS2b	a a . . . . c . . . . a . . t . . . . . . . . . . c . . . . . . . . . c . g . . . . . g . a - - . . . . . t . . . . . . . . . t g . c . c .	2	H25
WH1	a a . . . . c . . . . a . . t . . . . . . . . . . a . . . . . . . g . c . g . . . . . g . a . . . . . . . . . . . . . . . . . . g . . . c .	3	H26
WH2	a a . . . . c . . . . a . . t . . . . . . . . . . c . . . g . . . . t c . g . . . . . g . a . . . . . . . . . . . . . . . . . . g . . . c .	3	H27
WH4	a . . . . . . . . . . a . . . . - - . . . . . . . c . . . . . . g . . c . . . . . . . . . . . . . . . . . . . . . c . . . . . . g . . . . .	6	H28
AS	. . . . . . . . . . . . . . . . . . . . . . . . . . . . . . . . . . . . . . . . . . . . . . . . . . . . . . . . . c . . . . . . g . . . c .	15	H29

**Table 2:** The representative specimens fixed in hybrid genotypes from the putative parental sequences at four nucleotide positions in *rpS7*.

	Position		52	113	296	435
Parent	AS(5) *		T	C	T	A
	TH(5) *		T	C	T	A
Hybrid	JJ1	a	T	C	T	A
		b	A	A	C	C
	JJ6	a	T	C	T	A
		b	A	A	C	C
Hybrid	XS1	a	T	C	T	A
		b	A	A	C	C
	XS2	a	T	C	T	A
		b	A	A	C	C
Parent	PY(6) *		A	A	C	C

* The bracketed figure indicates the number of specimens. The lowercase stands for the different genotype at nucleotide positions from the same specimen.

**Table 3:** Summary statistics of *rpS7*, ITS1 and COI in *Coilia nasus*.

	Nucleotide Composition (%)		Variable Sites			Haplotypes		AMOVA (%)	
	
	A + T	G + C	single	parsi	indel	H	Hd	among	within
*rpS7*	56.5	43.5	35	26	9	29	0.76	29.9	70.1
ITS1	31.8	68.2	21	2	18	29	0.69	10.3	89.7
COI	55.5	45.5	4	6	0	9	0.68	1.9	98.1

	**Nucleotide Diversity**		**Trans/Transv**			**Neutrality Tests**			
	
	among	within	s	v	s/v	Taj D	P^*^	Fu	P^*^
	
*rpS7*	0.013–0.017	0.013	45	16	2.8	−1.328	N	−2.377	N
ITS1	0.003–0.009	0.006	16	9	1.8	−2.263	Y	−2.377	N
COI	0.001–0.005	0.003	8	2	4.0	−1.070	N	−1.018	N

*P* ^*^ denotes statistical significance (*P* < 0.05).

**Table 4:** Types and positions of the repeat motifs for ITS1 in *Coilia nasus*.

Repeats	Sequence Position	Region of Secondary Structure
(CT)^5–8^CC(CT)^3^	96–121	4th internal loop
(CT)^8^	96–111	4th internal loop
(CCAAA)^1–2^	281–297	2th internal loop

**Table 5:** PCR primers used for nuclear and mitochondrial data analysis.

Primer	Gene	Sequence (5′-3′)	Source
S7RPEX2R	*rpS7*-1	TGGCCTCTTCCTTGGCCGTC	Reference [6]
S7RPEX2R	*rpS7*-1	AACTCGTCTGGCTTTTCGCC	Reference [6]
ITS1F	ITS1	AGGTGAACCTGCGGAAGG	Present study
ITS2R	ITS1	TGATCCACCGCTAAGAGTTGTA	Present study
COIBF	COI	TGGCAATYACACGTTGATTYT	Present study
COIBR	COI	TTHCCBGCRTRRTARGCTACRA	Present study
